# Suppression of TAK1 pathway by shear stress counteracts the inflammatory endothelial cell phenotype induced by oxidative stress and TGF-β1

**DOI:** 10.1038/srep42487

**Published:** 2017-02-17

**Authors:** Ee Soo Lee, Llorenç Solé Boldo, Bernadette O. Fernandez, Martin Feelisch, Martin C. Harmsen

**Affiliations:** 1 University of Groningen, University Medical Center Groningen, Department of Pathology and Medical Biology, Groningen, NL-9713 GZ, The Netherlands; 2 University of Southampton, Southampton General Hospital, Faculty of Medicine, Clinical and Experimental Sciences, Southampton, SO166YD, United Kingdom

## Abstract

Endothelial dysfunction is characterised by aberrant redox signalling and an inflammatory phenotype. Shear stress antagonises endothelial dysfunction by increasing nitric oxide formation, activating anti-inflammatory pathways and suppressing inflammatory pathways. The TAK1 (MAP3K7) is a key mediator of inflammation and non-canonical TGF-β signalling. While the individual roles of TAK1, ERK5 (MAPK7) and TGF-β pathways in endothelial cell regulation are well characterised, an integrative understanding of the orchestration of these pathways and their crosstalk with the redox system under shear stress is lacking. We hypothesised that shear stress counteracts the inflammatory effects of oxidative stress and TGF-β1 on endothelial cells by restoring redox balance and repressing the TAK1 pathway. Using human umbilical vein endothelial cells, we here show that TGF-β1 aggravates oxidative stress-mediated inflammatory activation and that shear stress activates ERK5 signalling while attenuating TGF-β signalling. ERK5 activation restores redox balance, but fails to repress the inflammatory effect of TGF-β1 which is suppressed upon TAK1 inhibition. In conclusion, shear stress counteracts endothelial dysfunction by suppressing the pro-inflammatory non-canonical TGF-β pathway and by activating the ERK5 pathway which restores redox signalling. We propose that a pharmacological compound that abates TGF-β signalling and enhances ERK5 signalling may be useful to counteract endothelial dysfunction.

The vascular endothelium is a monolayer of cells that acts as the regulatory interface between blood and the vessel wall. Given the capability to receive and respond to biochemical as well as biomechanical stimuli, the endothelium is a key regulator of cardiovascular homeostasis^1^. Adverse alterations of the endothelial phenotype (endothelial dysfunction) precede the pathogenesis of cardiovascular disorders, particularly atherosclerosis^1^,^2^ and pulmonary hypertension^3^. The maintenance of a healthy endothelial phenotype relies on a delicate balance between nitric oxide (NO) production and reactive oxygen species (ROS) formation, both of which are crucial to the maintenance of cellular redox tone and the functioning of redox-related cell signalling. A decreased NO bioavailability, secondary to enhanced NO degradation by ROS can tip the redox balance and cause impaired NO-mediated signalling, an early hallmark of endothelial dysfunction^2^,^4^. In physiology, the phenotype of endothelial cells is tightly regulated by their responses to mechanical forces, especially shear stress^5^,^6^,^7^. Shear stress exerted by laminar blood flow increases NO bioavailability, while reducing ROS production. Therefore, shear stress safeguards endothelial redox homeostasis and counteracts endothelial dysfunction^5^,^7^,^8^.

The protective effects of shear stress on endothelial cells extend to its inhibition of inflammatory signalling cascades, such as nuclear factor kappa-light-chain-enhancer of activated B cell (NFκB)^5^,^9^ and p38 mitogen-activated protein kinase (MAPK)^10^ pathways. The expression of inflammatory entities, such as adhesion molecules and chemoattractants, that are activated by these signalling pathways, is also inhibited by shear stress^5^,^6^,^7^,^8^,^9^. Shear stress also elicits its protective effects through activation of mitogen-activated protein kinase 7 (MAPK7), also known as extracellular signal-regulated kinase 5 (ERK5)^7^. ERK5 signalling downregulates inflammatory entities through induction of the anti-inflammatory transcription factors, Kruppel-like factor 2 (KLF2)^11^ or KLF4^12^. Notably, TGF-β signalling also mediates shear-induced KLF2 expression through the activin receptor-like kinase 5 (ALK5)/SMAD pathway^13^,^14^. While the individual roles of NFkB, p38 MAPK, ERK5 and TGF-β pathways in endothelial dysfunction are well delineated, an understanding of the orchestration of these pathways and their crosstalk with the redox system in the context of relevant haemodynamic forces remain obscure. In addition to activating the canonical SMAD pathway, TGF-β also activates the non-canonical mitogen-activated protein kinase kinase kinase 7 (MAP3K7), also known as TGF-β-activated kinase 1 (TAK1) pathway^15^. Activation of TAK1 by inflammatory cytokines induces the expression of inflammatory entities in endothelial cells^9^. Surprisingly, the consequences of TAK1 activation for endothelial cells upon TGF-β stimulation and its regulation by shear stress remain unknown.

The levels of oxidative stress and TGF-β1 increase upon vascular damage^4^,^16^. However, the molecular mechanisms by which shear stress regulates the phenotype of endothelial cells upon oxidative stress and TGF-β1 stimulation are poorly understood. Our earlier studies revealed that shear stress suppresses a severe form of endothelial dysfunction, TGF-β-induced endothelial-to-mesenchymal transition (EndMT), through ERK5 activation^17^. Here, we hypothesised that shear stress counteracts the inflammatory effects of oxidative stress and TGF-β1 on endothelial cells by repressing the TAK1 pathway and by restoring redox balance. To test this hypothesis, we subjected human umbilical vein endothelial cells (HUVEC) to the pro-inflammatory (ROS) and pro-fibrotic (TGF-β1) triggers, and dissected the associated cell signalling responses of ERK5, ALK5 and TAK1 to shear stress using a combination of molecular biological, biochemical and pharmacological tools.

## Results

### TGF-β1 aggravates the inflammatory effects of oxidative stress

**Figure 1:**
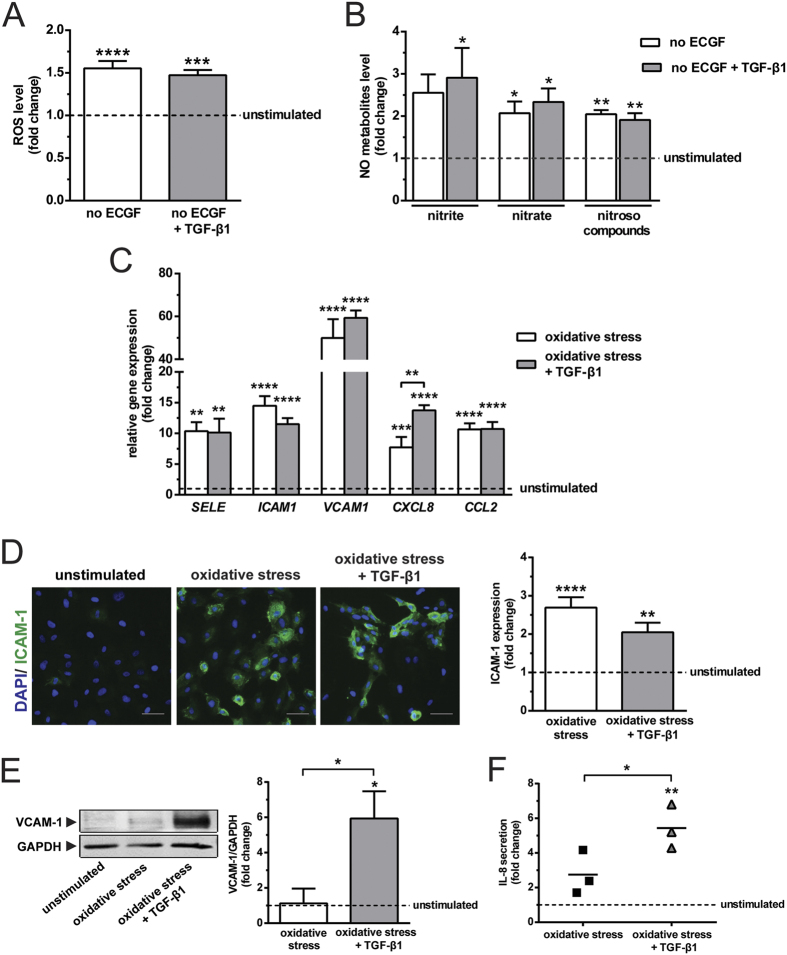
TGF-β1 aggravates the inflammatory effects of endothelial cell growth factor deprivation that induces oxidative stress. (**A**) Endothelial cell growth factor (ECGF) deprivation induces the formation of intracellular ROS (N = 3). TGF-β1 has negligible effects on the production of ROS (N = 3). (**B**) ECGF deprivation induces the formation of NO metabolites, nitrite, nitrate and nitroso compounds (N = 3). TGF-β1 has minimal effects on the production of NO metabolites (N = 3). (**C**) Oxidative stress induces the gene expression of adhesion molecules (*SELE, ICAM1* and *VCAM1*) and chemoattractants (*CXCL8* and *CCL2*) as compared with unstimulated condition (shown as a dotted line; N = 3). TGF-β1 augments the upregulation of *CXCL8* (N = 3). (**D**) Oxidative stress induces the protein expression of ICAM-1 (N = 3). TGF-β1 has little effect on the elevation of ICAM-1 expression (N = 3). Scale bar represents 50 μm. The combined effect of oxidative stress and TGF-β1 accentuates (**E**) the expression of VCAM-1 (N = 3) and (**F**) the secretion of IL-8 (N = 3). *p < 0.05, **p < 0.01, ***p < 0.001 & ****p < 0.0001.

Studies about the combined effects of bovine brain extract (referred to as endothelial cell growth factors, ECGF throughout the text) deprivation and TGF-β1 on redox balance and inflammation are scarce. Therefore, we investigated the influence of ECGF deprivation and TGF-β1 stimulation on ROS and NO metabolites formation, as well as the subsequent expression of inflammatory molecules by HUVEC. ECGF deprivation caused a 1.6-fold increase of intracellular ROS formation, but TGF-β1 stimulation did not further affect ROS induction (Fig. 1A). Interestingly, ECGF deprivation also increased NO production as evidenced by an elevation of nitrite, nitrate and nitroso compounds, whereas TGF-β1 had no added effect (Fig. 1B). Consistent with previous report^18^, these results demonstrate that increases in oxidative stress are counterbalanced by an up-regulation of endogenous NO production, and that TGF-β1 has insignificant influence on this redox reaction.

The oxidative stress caused by ECGF deprivation was associated with an increase in the expression of inflammatory molecules, *SELE* (10.3-fold), *ICAM1* (14.5-fold), *VCAM1* (50-fold), *CXCL8* (7.7-fold) and *CCL2* (10.7-fold; Fig. 1C). Of note, TGF-β1 caused an additional increase of *CXCL8* expression (Fig. 1C). Oxidative stress alone or in conjunction with TGF-β1 stimulation did not alter the expression of *TNFA* and *IL1B*. However, the combined stimulation with oxidative stress and TGF-β1 increased *IL6* expression by 1.7-fold (see Supplementary Fig. S1A). Oxidative stress induced the protein expression of ICAM-1 (2.7-fold), but there was no added effect of TGF-β1 on this induction (Fig. 1D). Of note, oxidative stress alone did not alter the expression VCAM-1, while oxidative stress together with TGF-β1 caused a 6-fold upregulation (Fig. 1E). TGF-β1 synergised with oxidative stress in inducing IL-8 secretion, as shown by the 2-fold higher induction of IL-8 secretion upon treatment with TGF-β1 compared to oxidative stress alone (Fig. 1F). These data indicate that dependent on the redox status of endothelial cells, the effects of TGF-β1 on inflammatory molecules expression are variable. Endothelial cells stimulated by oxidative stress and TGF-β1 had 27-fold higher interaction with leukocytes, as compared with the unstimulated control (see Supplementary Fig. S1B). Of note, this inflammatory endothelial phenotype was endowed with the feature of EndMT, as shown by the upregulation of mesenchymal markers, *ACTA2, TAGLN* and *CNN1,* as well as the downregulation of *PECAM1, THBD* and *NOS3* (see Supplementary Fig. S1C).

### Laminar shear stress suppresses the inflammatory effects of oxidative stress and TGF-β1

**Figure 2:**
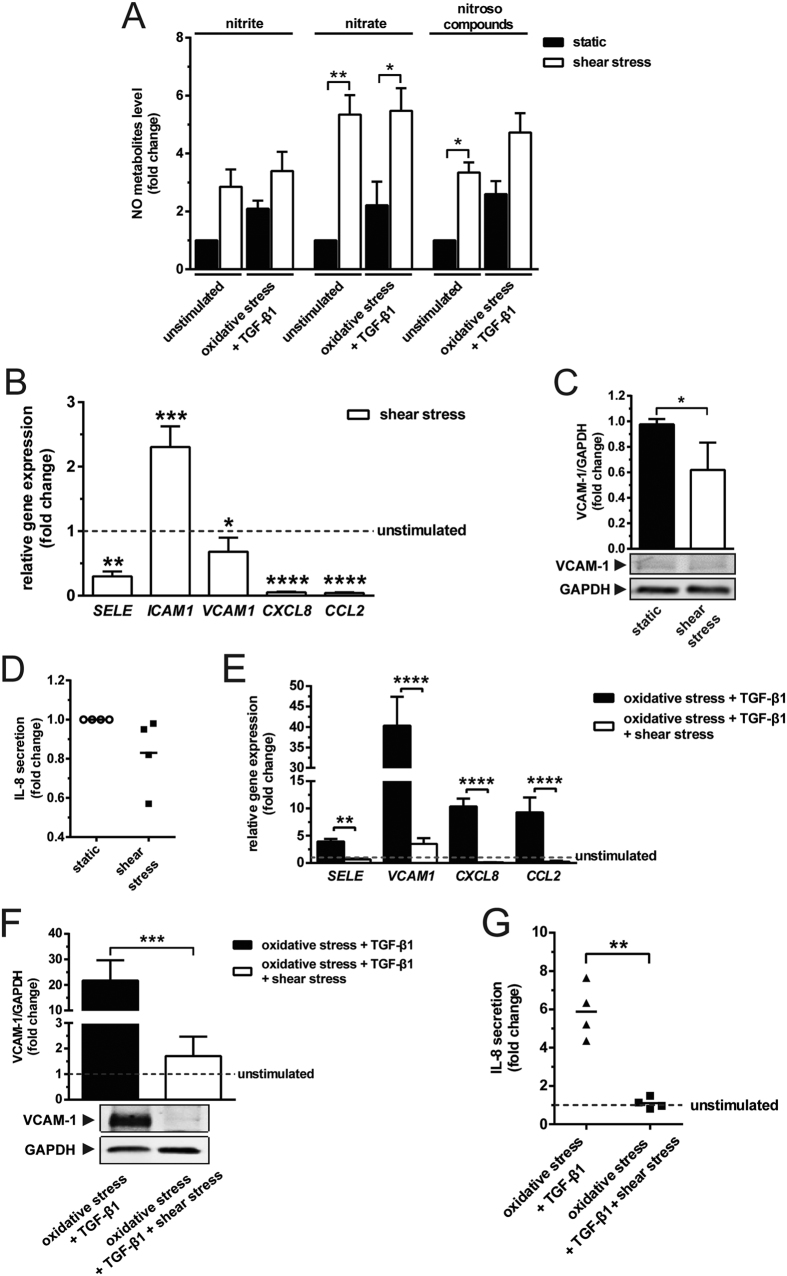
Shear stress preserves phenotype of endothelial cells. (**A**) Shear stress induces endothelial NO metabolites production, particularly nitrate and nitroso compounds (N = 3). (**B**) In comparison with the static condition (shown as a dotted line), shear stress inhibits the expression of *SELE, VCAM1, CXCL8* and *CCL2,* but induces the expression of *ICAM1* (N = 3). (**C**) Shear stress suppresses the protein expression of VCAM-1 as compared with the static condition (N = 6). (**D**) Shear stress has no effect on the secretion of IL-8 (N = 4). (**E**) Shear stress downregulates the elevated expression of *SELE, VCAM1, CXCL8* and *CCL2* (N = 3). Shear stress downregulates the increased expression of (**F**) VCAM-1 (N = 7) and (**G**) IL-8 (N = 4) resulted from oxidative stress and TGF-β1 stimulation. Data are presented relative to the unstimulated static condition (shown as a dotted line). *p < 0.05, **p < 0.01, ***p < 0.001 & ****p < 0.0001.

To assess the regulation of endothelial phenotype by shear stress, we exposed endothelial cells to laminar shear stress at a magnitude of 20 dynes/cm^2^. Shear stress enhanced endothelial NO production (Fig. 2A), down-regulated the expression of *SELE* (3.3-fold)*, VCAM1* (1.4-fold)*, CXCL8* (19.2-fold) and *CCL2* (23.8-fold), while up-regulated the expression of *ICAM1* (2.3-fold; Fig. 2B). In agreement with the gene expression data, shear stress downregulated VCAM-1 protein expression (Fig. 2C). IL-8 secretion did not change in response to shear stress (Fig. 2D).

Upon oxidative stress and additional TGF-β1 stimulation, sheared endothelial cells had a 6-, 11.5-, 94- and 42-fold downregulation of *SELE, VCAM1, CXCL8* and *CCL2* (Fig. 2E), respectively, when compared with the static control. Notably, shear stress repressed the upregulation of VCAM-1 (Fig. 2F) and IL-8 (Fig. 2G) protein expression by 12.7- and 5.3-fold, respectively. These data demonstrate that shear stress attenuates the combined inflammatory effects of oxidative stress and TGF-β1, and this effect is likely mediated via an increase in NO production.

### Activation of ERK5 reduces oxidative stress, but does not repress the inflammatory effects of TGF-β1

**Figure 3:**
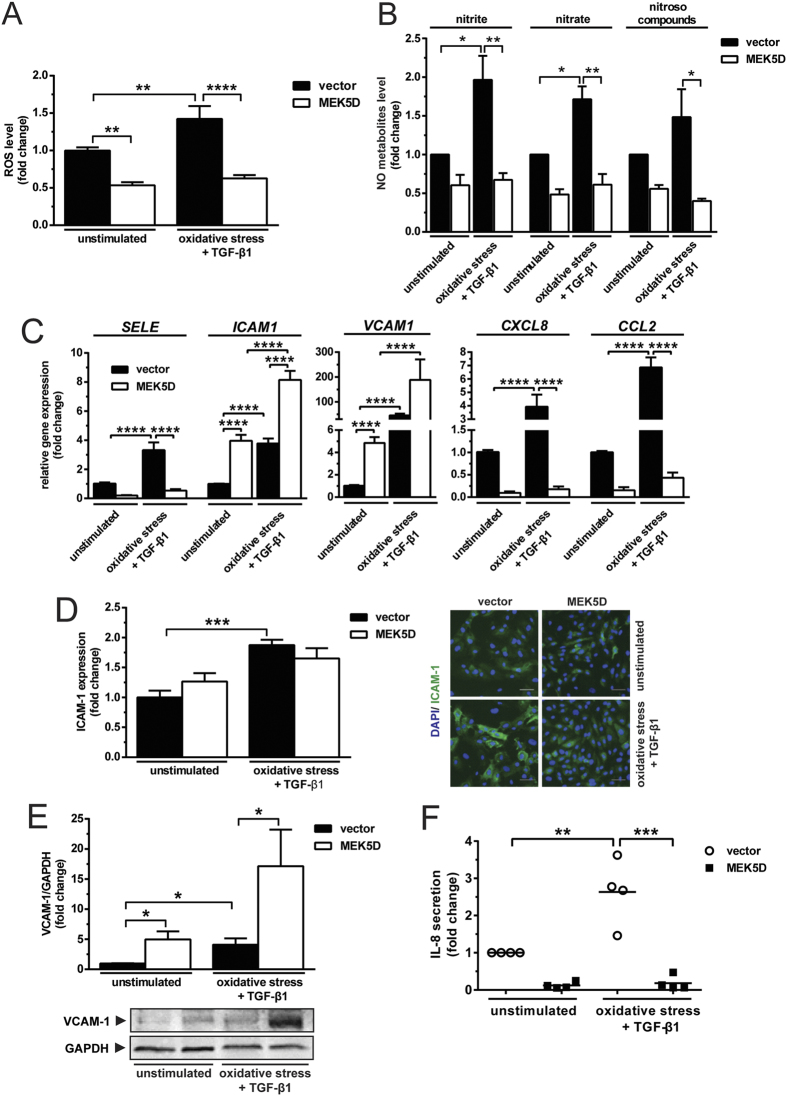
Endothelial cells stably expressing MEK5D are protected from oxidative stress, but have increased expression of ICAM-1 and VCAM-1 upon TGF-β1 stimulation. Endothelial cells transduced with a constitutively active mutant of MEK5 (MEK5D) produce lower level of (**A**) ROS and (**B**) NO metabolites than the vector control (N = 3). TGF-β1 does not alter the effects of ERK5 on downregulating the generation of ROS and NO metabolites (N = 3). (**C**) Activation of ERK5 pathway downregulates the induced expression of *SELE, CXCL8* and *CCL2*, but does not repress the elevation of *ICAM1* and *VCAM1* (N = 3). (**D**) ERK5 activation does not suppress the protein expression of ICAM-1 (N = 3). Scale bar represents 50 μm. (*E*) TGF-β1 intensifies the induced expression of VCAM-1 by ERK5 (N = 4). (**F**) Activation of ERK5 pathway suppresses the secretion of IL-8 (N = 4). *p < 0.05, **p < 0.01, ***p < 0.001 & ****p < 0.0001.

We were intrigued to elucidate as to how ERK5 signalling influences the combined effects of oxidative stress and TGF-β1, and vice versa in terms of endothelial phenotype regulation. To address this, we examined cellular redox state and phenotype of MEK5D-transduced cells subjected to oxidative stress and stimulated with TGF-β1. MEK5D is a constitutively active mutant of MEK5 that induces sustained activation of the ERK5 pathway^12^. Sustained activation of ERK5 suppressed the generation of both, ROS (Fig. 3A) and NO production (Fig. 3B). TGF-β1 did not alter the effects of ERK5 on maintenance of redox poise. Activation of ERK5 inhibited the expression of *SELE, CXCL8* and *CCL2*, but up-regulated the expression of *ICAM1* and *VCAM1* (Fig. 3C). Upon treatment with TGF-β1, expression of *ICAM1* and *VCAM1* in MEK5D-transduced cells were enhanced by 2- and 39-fold, respectively (Fig. 3C). Notably, TGF-β1 synergised with the ERK5-mediated upregulation of *ICAM1* and *VCAM1*. In spite of the enhanced transcript expression, TGF-β1 had negligible effect on protein expression of ICAM-1 in MEK5D-transduced cells (Fig. 3D). Activation of ERK5 induced the expression of VCAM-1 by a factor of 5 (Fig. 3E). Remarkably, upregulation of VCAM-1 was further enhanced (3.5-fold) when ERK5 pathway was activated upon TGF-β1 stimulation (Fig. 3E). The TGF-β-induced IL-8 secretion was strongly inhibited (14.6-fold) when MEK5D was stably expressed (Fig. 3F). Collectively, our data indicate that despite the marked modulatory effects of ERK5 pathway on the magnitude of combined ROS and NO production, it only partially rescues the TGF-β1-induced alterations in endothelial phenotype.

### Shear stress antagonises the activation of canonical TGF-β signalling, independent of ERK5

**Figure 4:**
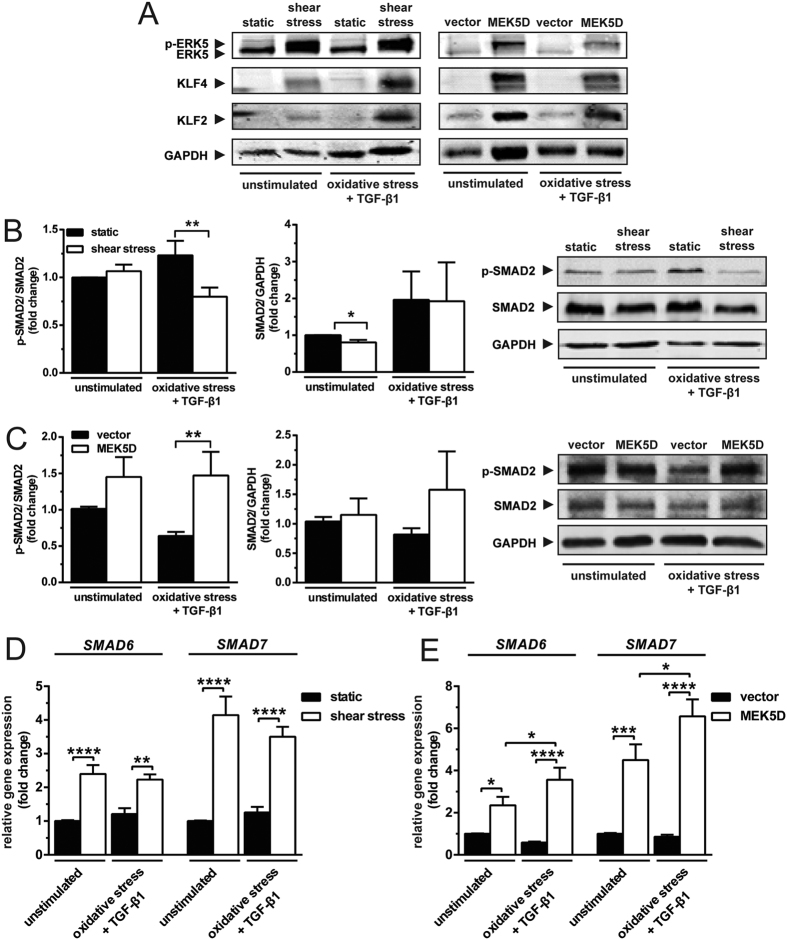
Shear stress abates the activation of canonical TGF-β signalling, independent of the ERK5 pathway. (**A**) Immunoblot analyses of ERK5 activation and the expression of KLF2 and KLF4 induced by shear stress and MEK5D mutant (representative blots are shown). (**B**) Shear stress attenuates the phosphorylation of SMAD2 induced by TGF-β1. This suppression is not caused by the reduced level of total SMAD2 (N = 6). (**C**) ERK5 activation under static condition does not suppress the SMAD2 activation. ERK5 activation has no effect on the expression of total SMAD2 (N = 4). (**D**) Shear stress upregulates the expression of *SMAD6* and *SMAD7*. TGF-β1 has no effects on these upregulations (N = 4). (**E**) ERK5 activation under static condition upregulates the expression of *SMAD6* and *SMAD7*. TGF-β1 has no effects on these upregulations (N = 2). *p < 0.05, **p < 0.01, ***p < 0.001 & ****p < 0.0001.

Under stimulation with TGF-β1, shear stress repressed, whereas ERK5 signalling augmented the expression of VCAM-1. These differences prompted us to dissect the underlying mechanisms. In spite of the TGF-β1 stimulation, both sheared and MEK5D-transduced cells showed increased ERK5 phosphorylation, as well as enhanced KLF2 and KLF4 expression (Fig. 4A). Others reported that KLF2 attenuates canonical TGF-β signalling through reduction of SMAD2 phosphorylation and inhibition of SMAD3/4 transcriptional activity^19^. We therefore investigated whether shear stress downregulates canonical TGF-β signalling while induces KLF2 expression via ERK5 activation. Our data show that TGF-β1 stimulation induced SMAD2 phosphorylation, an effect that was repressed approximately 1.5-fold by shear (Fig. 4B). This repression was not caused by the reduced expression of total SMAD2 (Fig. 4B). Activation of ERK5 signalling under static conditions did not suppress the phosphorylation of SMAD2 (Fig. 4C). Upon stimulation with TGF-β1, MEK5D-transduced cells showed a 2.3-fold higher phosphorylation of SMAD2 than the vector controls (Fig. 4C), implying that the TGF-β signalling was reinforced by activation of the ERK5 pathway, the mechanism of which is unclear. Sustained ERK5 activation did not alter the expression of total SMAD2 (Fig. 4C). Furthermore, there was a 4-fold increased expression of SMAD2 target gene, *TAGLN* in MEK5D-transduced cells under stimulation with TGF-β1 (see Supplementary Fig. S2). These results demonstrate that shear stress suppresses, whereas ERK5 signalling augments the activation of canonical TGF-β signalling.

Shear stress induced the expression of the TGF-β canonical inhibitors, *SMAD6* and *SMAD7* 2.4- and 4.1-fold (Fig. 4D), respectively. Shear-induced *SMAD6* and *SMAD7* expression was not altered by TGF-β1 stimulation (Fig. 4D). In concordance with shear stress, constitutive activation of ERK5 (MEK5D-transduced cells) under static condition enhanced the expression of *SMAD6* and *SMAD7* 2.4- and 4.5-fold, respectively. Intriguingly, TGF-β1 stimulation augmented the upregulation of *SMAD6* and *SMAD7* in MEK5D-transduced cells (Fig. 4E), supporting the notion that ERK5 signalling enhances the activation of TGF-β signalling. These data show that both shear stress and activation of ERK5 induce the expression of inhibitory SMADs (I-SMADs), *i.e. SMAD6* and *SMAD7*.

### Inhibition of canonical TGF-β signalling suppresses oxidative stress, but does not influence the expression of ICAM-1, VCAM-1 and IL-8

**Figure 5:**
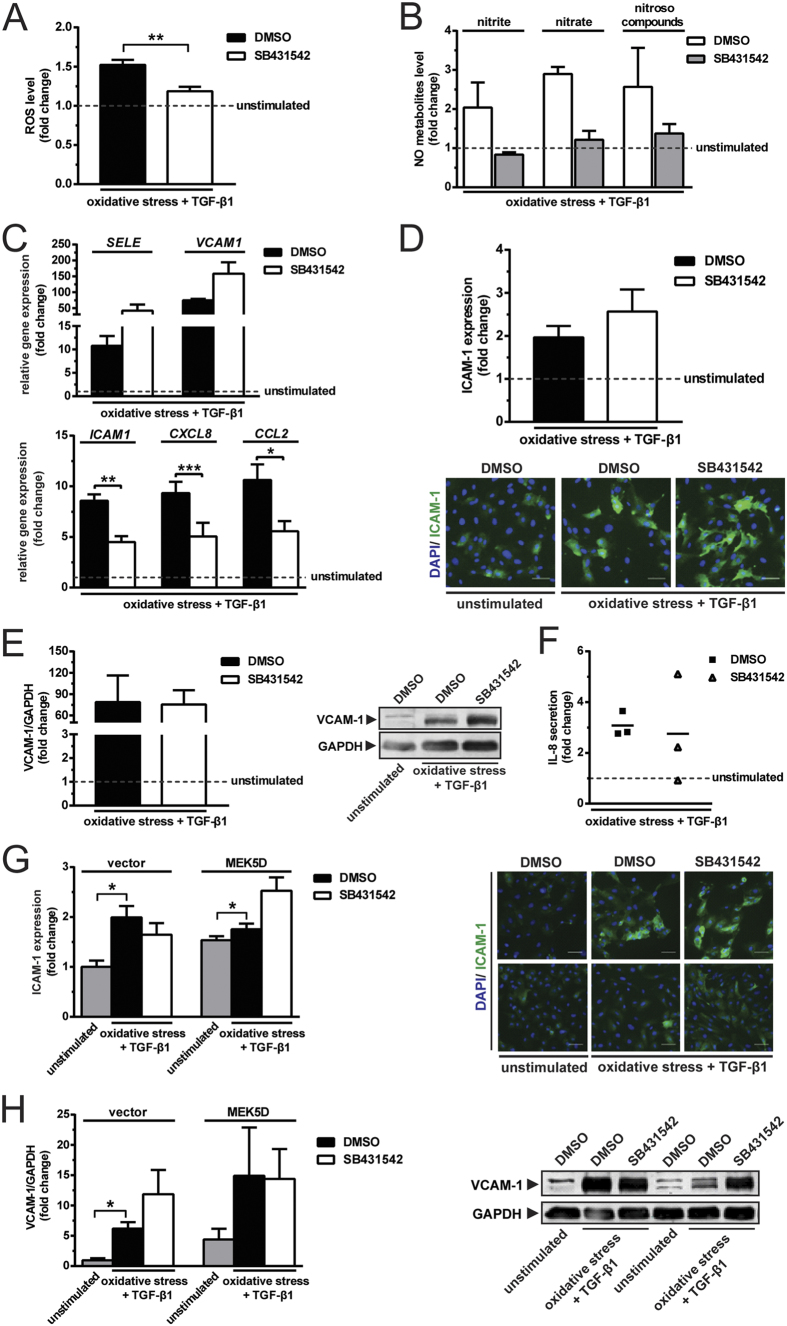
Inhibition of canonical TGF-β signalling reduces oxidative stress, but does not influence the expression of ICAM-1, VCAM-1 and IL-8. Attenuation of ALK5 kinase activity reduces the level of (**A**) ROS (N = 3), but has insignificant effects on (**B**) NO metabolites production (N = 3). (**C**) ALK5 inhibition antagonises the induced expression of *ICAM1, CXCL8* and *CCL2*, but not *SELE* and *VCAM1*. ALK5 inhibition does not repress the induced protein level of (**D**) ICAM-1, (**E**) VCAM-1 and (**F**) IL-8 (N = 3). The enhanced expression of (**G**) ICAM-1 and (**H**) VCAM-1 in MEK5D-transduced cells remains unaltered despite the inhibition of ALK5 (N = 3). Scale bar represents 50 μm. *p < 0.05, **p < 0.01 & ***p < 0.001.

Since shear stress represses the activation of TGF-β signalling, we inhibited the canonical ALK5/SMAD pathway with the ALK5 inhibitor, SB431542, to investigate whether canonical TGF-β signalling affects redox balance and endothelial phenotype. Inhibition of ALK5/SMAD signalling reduced ROS formation (1.3-fold; Fig. 5A), but had insignificant effect on NO production (Fig. 5B). ALK5/SMAD signalling had no effect on the expression of *SELE* and *VCAM1* (Fig. 5C). Notably, *ICAM1, CXCL8* and *CCL2* were almost 2-fold downregulated (Fig. 5C). Inhibition of the ALK5/SMAD signalling had no effects on the induced expression of ICAM-1 (Fig. 5D), VCAM-1 (Fig. 5E) and IL-8 (Fig. 5F) upon TGF-β1 stimulation. Similarly, the expression of ICAM-1 (Fig. 5G) and VCAM-1 (Fig. 5H) in MEK5D-transduced cells was unaffected by the inhibition of ALK5. These data implicate a role for ALK5/SMAD signalling in ROS generation, but not in NO production and protein expression of inflammatory molecules.

### Inhibition of TAK1 pathway or mitochondrial ROS production suppresses the upregulation of ICAM-1, VCAM-1 and IL-8

**Figure 6:**
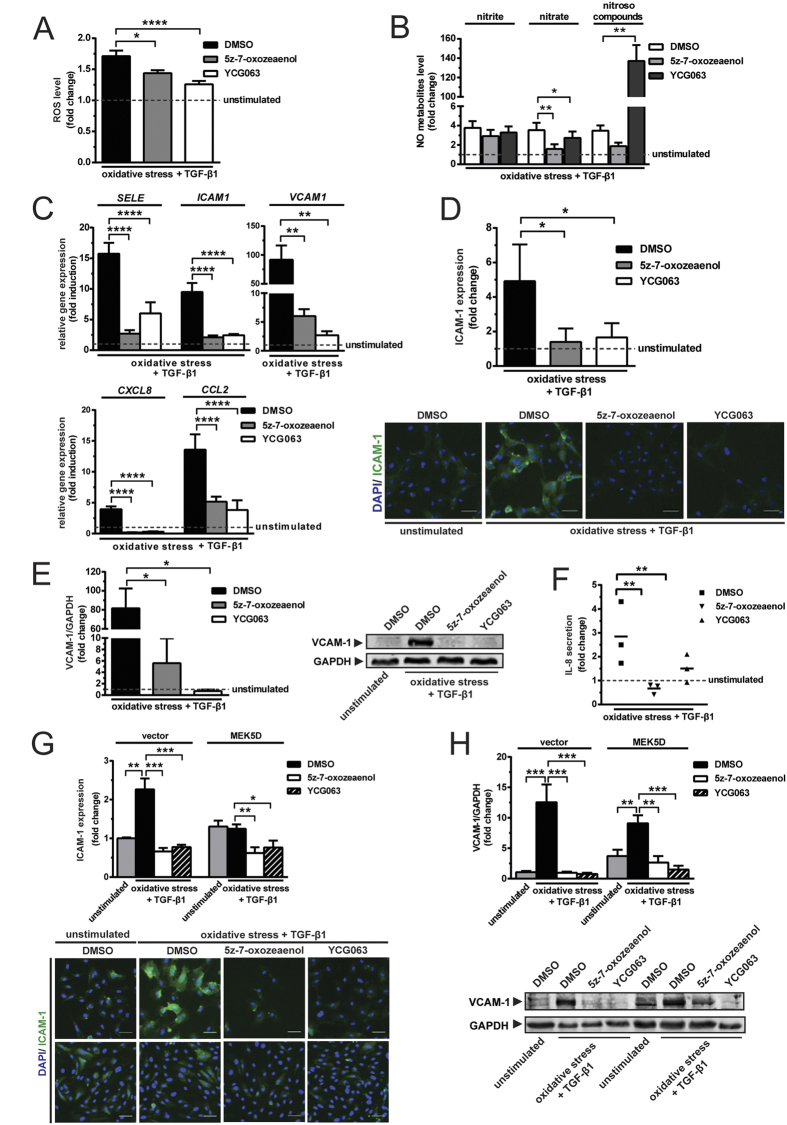
Suppression of either TAK1 activation or mitochondrial ROS production represses the upregulation of inflammatory molecules. (**A**) Both 5z-7-oxozeaenol and YCG063 reduce the production of ROS (N = 3). (**B**) 5Z-7-oxozeaenol downregulates the nitrate production, but has little effect on the generation of nitrite and nitroso compounds. YCG063 induces the formation nitroso compounds potently, reduces the nitrate production and has no effect on the nitrite production (N = 3). Inhibition of TAK1 or suppression of mitochondrial ROS generation counteracts the induced expression of (**C**) *SELE, ICAM1, VCAM1, CXCL8* and *CCL2*, as well as (**D**) ICAM-1, (**E**) VCAM-1 and (**F**) IL-8 (N = 3). The induced expression of (**G**) ICAM-1 and (**H**) VCAM-1 in MEK5D-transduced endothelial cells are repressed by either 5z-7-oxozeaenol or YCG063 (N = 3). Scale bar represents 50 μm. *p < 0.05, **p < 0.01, ***p < 0.001 & ****p < 0.0001.

Because the inhibition of canonical ALK5/SMAD signalling did not suppress the induced expression of ICAM-1, VCAM-1 and IL-8, we hypothesised that shear stress inhibits the expression of these molecules by regulating either the non-canonical TAK1 pathway or redox poise. The TAK1 inhibitor, 5z-7-oxozeaenol and the mitochondrial ROS inhibitor, YCG063, both reduced the formation of intracellular ROS by 1.2- and 1.4- fold, respectively (Fig. 6A). Inhibition of TAK1 and mitochondrial ROS reduced nitrate generation by 2.2- and 1.3-fold, respectively, but had insignificant effects on nitrite formation (Fig. 6B). Of note, the production of nitrosated species increased dramatically (more than 30-fold) as a result of mitochondrial ROS inhibition, in the presence (see Supplementary Fig. S3) and absence (Fig. 6B) of constitutive ERK5 activation; these effects were not accompanied by prominent enhanced nitrite/nitrate formation, indicating they were not caused by increased NO production. While both pathway of formation and functional significance of this finding remain unclear at present, this data are testimony to marked alterations in mitochondrial nitrosative stress triggered by YCG063.

5Z-7-oxozeaenol suppressed the induced expression of *SELE, ICAM1, VCAM1, CXCL8* and *CCL2* by 5.8-, 4.5-, 15.3-, 20.5-, 2.6-fold, respectively (Fig. 6C). Similarly, YCG063 also downregulated the induced expression of *SELE, ICAM1, VCAM1, CXCL8, CCL2* by 2.6-, 3.9-, 33.9-, 12.7-, 3.5-fold, respectively (Fig. 6C). Treatment with 5z-7-oxozeaenol or YCG063, downregulated the induction of ICAM-1 by 3-fold (Fig. 6D). The increased level of VCAM-1 was downregulated by 14.6- and 113.2-fold in response to the treatment with 5z-7-oxozeaenol and YCG063, respectively (Fig. 6E). Inhibition of TAK1 or mitochondrial ROS formation resulted in a 1.9- and 4.3-fold reduction in the level of IL-8 secretion (Fig. 6F). Under the treatment with 5z-7-oxozeaenol or YCG063, the ICAM-1 expression of MEK5D-transduced cells were repressed almost 2-fold (Fig. 6G). VCAM-1 expression of MEK5D-transduced cells was downregulated by 3.4- and 6.1-fold in response to 5z-7-oxozeaenol or YCG063 treatment, respectively (Fig. 6H). Taken together, these data show that TAK1 or generation of mitochondrial ROS, or both contribute to oxidative stress and cause upregulation of inflammatory molecules. Interestingly, 5z-7-oxozeaenol or YCG063 repressed the upregulation of smooth muscle 22α (SM22α) (see Supplementary Fig. S4), suggesting that the TAK1 pathway and ROS contribute to induction of EndMT^20^,^21^ while exerting inflammatory effects on endothelial cells.

### Inhibition of the p38 MAPK or NFκB pathway suppresses inflammatory endothelial phenotype

**Figure 7:**
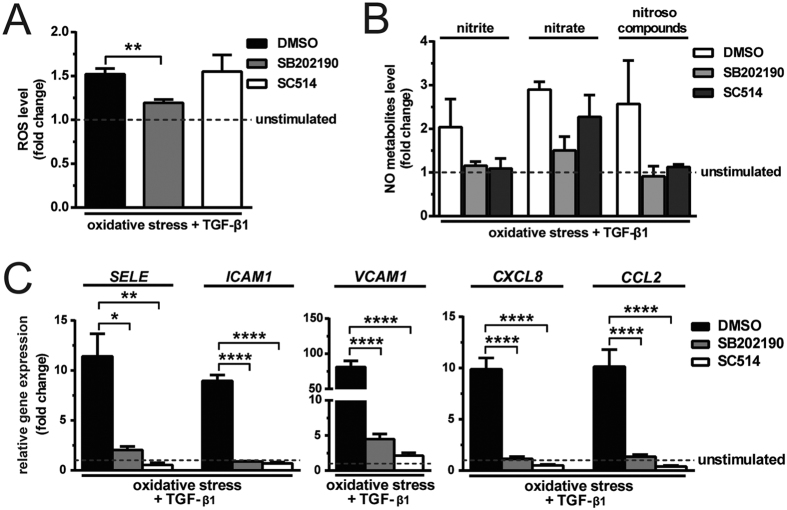
Inhibition of either p38 MAPK or NFκB pathway suppresses the upregulation of inflammatory molecules. (**A**) P38 MAPK inhibition reduces ROS production. NFκB pathway inhibition has no effect on ROS production. (N = 3) (**B**) Both SB202190 and SC514 have insignificant effect on NO metabolites production (N = 3). (**C**) Inhibition of either p38 MAPK or NFκB pathway suppresses the induced expression of *SELE, ICAM1, VCAM1, CXCL8* and *CCL2* (N = 3). *P < 0.05, **P < 0.01 & ****P < 0.0001.

Finally, we dissected the signalling cascades that act downstream of TAK1 and their attenuation of oxidative stress and suppression of endothelial inflammation. Endothelial cells subjected to oxidative stress and stimulated with TGF-β1, were treated with the respective inhibitor of p38 MAPK, SB202190, and of IKKβ, SC514. Inhibition of p38 MAPK decreased the induced formation of intracellular ROS by 1.3-fold. Inhibition of IKKβ had no effect on ROS formation (Fig. 7A). Inhibition of p38 MAPK or IKKβ had insignificant effect on NO production (Fig. 7B). Inhibition of p38 MAPK decreased the induced expression of *SELE, ICAM1, VCAM1, CXCL8* and *CCL2* by 5.6-, 10.2-, 18.1-, 8.7- and 7.4-fold (Fig. 7B), respectively. Similarly, IKKβ inhibition also decreased the induced expression of *SELE* (21.5-fold)*, ICAM1* (13-fold)*, VCAM1* (38-fold)*, CXCL8* (20-fold) and *CCL2* (26-fold; Fig. 7C). Taken together, these data suggest that TAK1 activation preferentially activates p38 MAPK or NFkB, or both, upon TGF-β1 stimulation to induce an inflammatory endothelial phenotype.

## Discussion

**Figure 8:**
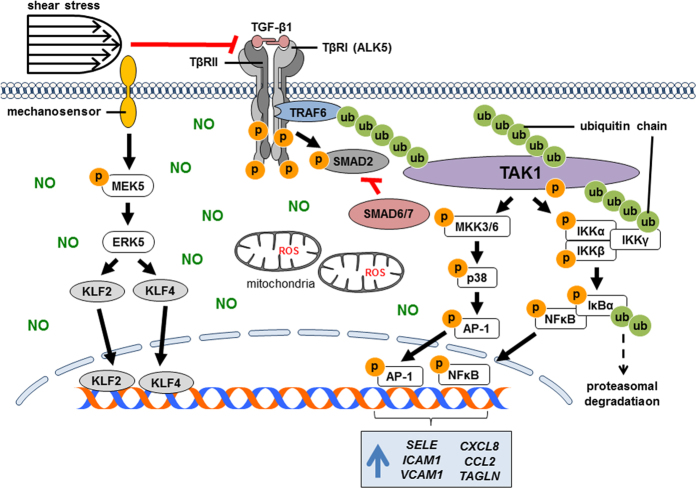
Proposed mechanosignalling crosstalk for regulation of endothelial phenotype by shear stress upon oxidative stress and TGF-β1 stimulation. ROS derived from mitochondria reduce NO bioavailability, impair NO-mediated signalling and induce the activation of inflammatory signalling, such as NFκB and p38 MAPK pathways. Shear stress attenuates ROS generation and increases NO bioavailability via the ERK5 pathway. Shear stress represses TGF-β1-induced inflammatory activation by suppressing p38 MAPK and NFκB signalling which act downstream of TAK1, in a manner independent of ERK5, redox poise and ALK5 kinase activity. Shear stress may attenuate the canonical ALK5/SMAD pathway via the upregulation of inhibitory SMADs, i.e. SMAD6 and SMAD7.

In this study, we dissected the interplay of different components in the network of mechanotransduction which suppresses the inflammatory endothelial phenotype. We showed that shear stress-activated ERK5 signalling restores the redox state of endothelial cells by adjusting NO and ROS production, but fails to antagonise the inflammatory activation by TGF-β1. Notably, high shear stress counteracts TGF-β1-induced inflammation by suppressing the non-canonical TGF-β pathway via TAK1, in an ERK5-independent manner. This indicates that although shear stress-activated ERK5 signalling restores the redox state of endothelial cells, it fails to antagonise the inflammatory effects of TGF-β1. Furthermore, we showed that shear stress abates the kinase activity of ALK5. By contrast, in the absence of shear stress, constitutive activation of ERK5 with MEK5D, augmented ALK5 activity while suppressing both, ROS and NO production. This suggests that suppression of TGF-β signalling by shear stress is independent of the ERK5 pathway and redox state (Fig. 8).

Our study suggests that oxidative stress not only contributes to impaired NO bioactivity by scavenging NO, but that rates of endothelial ROS and NO production are indeed coupled to maintain proper functioning of redox-related signalling including phosphorylation processes; thus, NO production not only compensatorily increased upon enhanced oxidative stress, but decreased proportionally upon repression of cellular ROS production. Reaction of ROS with NO lead to generation of pro-oxidants, such as peroxynitrite^4^,^22^. Peroxynitrite and other reactive species can alter endothelial phenotype by disrupting cellular redox signalling, as well as by inducing the activation of inflammatory pathways, such as NFκB and p38 MAPK^4^ (Fig. 8).

Our study demonstrated that TGF-β1 aggravates the inflammatory effects of oxidative stress via the non-canonical TGF-β pathway. Consistent with others, we showed that activation of ERK5 repressed the induction of *SELE*^23^*, CCL2* and IL-8^12^. Intriguingly, upon TGF-β1 stimulation, ERK5 signalling did not inhibit VCAM-1 gene and protein expression which decreased when the TAK1 signalling axis was inhibited. AMP-activated protein kinase (AMPK) mediates shear stress-induced ERK5 signalling, while increasing NO bioavailability and reducing oxidative stress^24^. This mechanistic evidence and our finding link shear stress and the ERK5 pathway with redox homeostasis. We suggest that shear stress represses oxidative stress-induced inflammation by restoring the redox state via ERK5 pathway, but the suppression of TGF-β1-induced inflammation depends on inactivation of TAK1 signalling (Fig. 8). A selective inhibition of either NFκB or p38 MAPK downregulated the expression of inflammatory molecules. This coincides with earlier findings that TAK1 requires NFκB^25^ and p38 MAPK^26^ to elicit its downstream effects on endothelial cells (Fig. 8). Activation of TAK1 depends predominantly on the affinity of TNF receptor-associated factor 6 (TRAF6) to TGF-β receptor I (TβRI), such as ALK5 and stimulation of TGF-β receptor II (TβRII) by TGF-β^27^,^28^. Therefore, we postulate that shear stress suppresses the activation of TAK1 and its downstream pathways by interfering either with the affinity of TRAF6 to ALK5 or with the binding of TGF-β1 to TβRII (Fig. 8).

The inhibitory SMADs (SMAD6 and SMAD7) are negative feedback regulators of the TGF-β signalling^29^. Earlier study elucidated KLF2 via upregulation of SMAD7, represses phosphorylation of SMAD2^19^. In our study, shear stress-induced *SMAD6* and *SMAD7* expression correlated with the downregulation of SMAD2 phosphorylation, which suggests that shear stress inhibits TGF-β signalling via inhibitory SMADs. Notably, SMAD2 phosphorylation was not attenuated in our MEK5D-transduced cells, despite the enhanced expression of KLF2, *SMAD6* and *SMAD7*. The overexpression of a SMAD2 target gene, *TAGLN* upon TGF-β stimulation in our study explains that the transcriptional activity of SMAD2 in MEK5D-transduced cells was conserved. These data suggest that shear stress inactivates TGF-β signalling in an ERK5-independent manner. We postulate that SMAD6 and SMAD7 proteins in MEK5D-transduced cells may have been degraded due to SUMO(small ubiquitin-like modifier)ylation and ubiquitylation upon TGF-β stimulation^30^. Alternatively, the post-transcriptional processes of these inhibitory *SMADs* may be affected by microRNAs^31^.

Collectively, the present study provides compelling evidence that regulation of the endothelial phenotype by shear stress involves an intricate crosstalk of multiple signalling axes, rather than an alteration of select individual pathways (Fig. 8). Our previous findings elucidated that shear stress mediates EndMT through the ERK5 pathway^17^. Here, we show that parallel to signalling through ERK5, which safeguards redox homeostasis, shear stress antagonises the inflammatory effects of TGF-β1 by suppressing the TAK1 pathway (Fig. 8), suggesting that shear stress counteracts endothelial dysfunction by activating ERK5 pathway and suppressing TGF-β pathway. We propose that the protective effects of shear stress on endothelial cells can be substituted by a pharmacological compound that enhances ERK5 signalling and abates TGF-β signalling concurrently.

## Materials and Methods

Extended materials and methods are available in supplementary information.

### Cell culture, stimulation and inhibition

HUVEC were obtained from Lonza (Breda, The Netherlands) and the Endothelial Cell Facility of University Medical Center Groningen, The Netherlands. Cells were maintained in endothelial cell medium, composed of, RPMI 1640 basal medium (Lonza, Verviers, Belgium), supplemented with 20% heat-inactivated foetal bovine serum (Invitrogen/GIBCO, CA, USA), 50 μg/ml endothelial cell growth factors supplement (bovine brain extract; homemade), 1% penicillin-streptomycin (Sigma-Aldrich, MA, USA), 2 mM L-glutamine (Lonza) and 5 U/ml heparin (Leo Pharma, Ballerup, Denmark). Cells were used for experiments at passage 6 and 7.

Confluent monolayers of HUVEC were stimulated for 48 h with or without 5 or 10 ng/ml citric acid activated-TGF-β1 (Peprotech, NJ, USA; #100–21 C) in RPMI 1640 basal medium, supplemented with 20% heat-inactivated foetal bovine serum, 2 mM L-glutamine, 5 U/ml heparin and 1% penicillin-streptomycin. Cells treated with endothelial cell medium were harvested as unstimulated controls, Cells were treated with pharmacological inhibitors for 48 h to inhibit desired signalling pathways.

### Shear stress experiments

Confluent monolayer of HUVEC in flow channel, μ-Slide I^0.4^ Luer (ibidi, Martinsried, Germany) were exposed to 20 dynes/cm^2^ shear stress for 48 h in endothelial cell medium or RPMI 1640 basal medium, supplemented with 5 or 10 ng/ml TGF-β1, 20% heat-inactivated foetal bovine serum, 2 mM L-glutamine, 5 U/ml heparin and 1% penicillin-streptomycin. Ibidi Pump System was employed for generation of laminar flow. Shear stress experiments were performed in an incubator at 37 °C with 5% CO_2_. Cells treated with the same media without exposure to flow were harvested as static controls.

### Retroviral transduction

A retroviral construct for stable expression of constitutively active rat MEK5-α1 (MEK5D) was generated as described before^12^. Retroviral transduction of HUVEC was performed as previously described^17^. Expanded transduced cells were seeded for experiments as described for wild type cells.

### ROS measurement

Cells were treated with 20 μM of 2′,7′-dichloroflorescein diacetate (Sigma-Aldrich; D6883) and analysed with FACSCalibur^TM^ flow cytometer (BD Biosciences, NJ, USA) at λmax, 520 nm. Average intensity was obtained by subtracting the mean fluorescent intensity of unstained samples from stained samples. Data of each experimental condition are presented as fold changes in average intensity relative to their respective experimental controls.

### Quantification of nitrite, nitrate and total nitroso compounds

Cellular formation of NO was quantified by the accumulation of nitrite, nitrate and total nitroso compounds in the cell culture media. The level of nitrite and nitrate in aliquots of the media was determined using a dedicated analysis system (ENO-20, EiCom, Japan), as previously described^32^. The level of nitroso compounds, comprising N-nitrosamines and S-nitrosothiols in aliquots of the same media was quantified by gas-phase chemiluminescence, as previously described^33^. Concentration of nitrite (μM), nitrate (μM) and nitroso compounds (nM) in each media was normalized to the respective numbers of cell in each experimental condition. Normalised data are presented as fold changes in concentration relative to experimental controls.

### RNA isolation and RT-qPCR

RNA was isolated with either TRIzol reagent (Invitrogen Corp, CA, USA) or RNA-Bee (Bio-Connect, The Netherlands) according to the manufacturer’s protocol. Primer sets (see Supplementary Table S1) were used to detect amplimers of interest. Data of each experimental condition are presented as fold changes in gene expression relative to their respective experimental controls.

### Immunofluorescent staining

Mouse anti-human ICAM-1 antibody (hybridoma supernatant; Hu5/3; kindly provided by Professor Dr. Michael A. Gimbrone Jr, Harvard Medical School, Boston, MA, USA)^34^,^35^ and SM22α antibody (1:200; Abcam, Cambridge, UK; ab14106) were used. Stained slides were analysed using TissueFaxs^®^ Zeiss AxioObserver Z1 Microscope System (TissueGnostics, Vienna, Austria). Mean fluorescent intensity quantification was performed with TissueQuest fluorescence analysis software (TissueGnostics). At least 500 cells were counted for each experimental condition. Data of each experimental condition are presented as fold changes in expression relative to their respective experimental controls.

### Immunoblotting

Odyssey Immunoblotting System (Li-COR Biosciences, USA) was employed. Antibodies against human VCAM-1 (1:100; Santa Cruz Biotechnology, USA; sc-8304), ERK5 (1:500; Upstate Cell Signaling Solutions, USA; #07–039), KLF4 (1:500; Santa Cruz Biotechnology; sc-20691), KLF2 (1:500; Santa Cruz Biotechnology; sc-28675), p-SMAD2 (Ser465/467; 1:200; Cell Signaling Technology, USA; #3108), SMAD2/3 (1:200; Cell Signaling Technology; #3102) and GAPDH (1:2000; Abcam, UK; ab9484) were used for detection of target proteins. Expression of target proteins was normalised to loading control, GAPDH. Data of each experimental condition are presented as fold changes in expression relative to their respective experimental controls.

### Sandwich enzyme-linked immunosorbent assay (ELISA)

Culture media of different experimental condition were collected. Concentration of IL-8 in media was quantified with Human IL-8 ELISA MAX^TM^ Standard Sets (BioLegend Inc, CA, USA) according to the manufacturer’s protocol. Concentration of IL-8 in each media was normalized to their respective numbers of cell in each experimental condition. Data of each experimental condition are presented as fold changes in IL-8 concentration (pg/ml) relative to their respective experimental controls.

### Statistical Analysis

All experimental data were obtained from two to seven independent experiments with duplicates or triplicates. All data are presented as means + standard error of the mean (SEM). Statistical analyses were performed with GraphPad Prism (Version 6.01; GraphPad Software, Inc., USA). For two-group comparisons, two-tailed ratio paired t test was performed. For multiple group comparisons, one-way analysis of variance (ANOVA) followed by Sidak’s post-test were carried out. For multiple categorical group comparisons, two-way ANOVA followed by Sidak’s post-test were executed. All statistical analyses were performed at the 95% of confidence interval. Differences between means were considered significant when probabilities (P) were less than 0.05.

## Additional Information

**How to cite this article**: Lee, E. S. *et al*. Suppression of TAK1 pathway by shear stress counteracts the inflammatory endothelial cell phenotype induced by oxidative stress and TGF-β1. *Sci. Rep.* **7**, 42487; doi: 10.1038/srep42487 (2017).

**Publisher's note:** Springer Nature remains neutral with regard to jurisdictional claims in published maps and institutional affiliations.

## Supplementary Material

Supplementary Information

## References

[b1] Deanfield J. E., Halcox J. P. & Rabelink T. J. Endothelial function and dysfunction: testing and clinical relevance. Circulation 115, 1285–1295 (2007). 17353456 10.1161/CIRCULATIONAHA.106.652859

[b2] Cai H. & Harrison D. G. Endothelial dysfunction in cardiovascular diseases: the role of oxidant stress. Circ Res 87, 840–844 (2000). 11073878 10.1161/01.res.87.10.840

[b3] Lewis L. J., Hoak J. C., Maca R. D. & Fry G. L. Replication of human endothelial cells in culture. Science 181, 453–454 (1973). 4718112 10.1126/science.181.4098.453

[b4] Pacher P., Beckman J. S. & Liaudet L. Nitric oxide and peroxynitrite in health and disease. Physiol Rev 87, 315–424 (2007). 17237348 10.1152/physrev.00029.2006 PMC2248324

[b5] Hahn C. & Schwartz M. A. Mechanotransduction in vascular physiology and atherogenesis. Nat Rev Mol Cell Biol 10, 53–62 (2009). 19197332 10.1038/nrm2596 PMC2719300

[b6] Zhou J., Li Y.-S. & Chien S. Shear stress–initiated signaling and its regulation of endothelial function. Arterioscler Thromb Vasc Biol 34, 2191–2198 (2014). 24876354 10.1161/ATVBAHA.114.303422 PMC4169328

[b7] Berk B. C. Atheroprotective signaling mechanisms activated by steady laminar flow in endothelial cells. Circulation 117, 1082–1089 (2008). 18299513 10.1161/CIRCULATIONAHA.107.720730

[b8] Hsieh H.-J., Liu C.-A., Huang B., Tseng A. & Wang D. Shear-induced endothelial mechanotransduction: the interplay between reactive oxygen species (ROS) and nitric oxide (NO) and the pathophysiological implications. J Biomed Sci 21, 3 (2014). 24410814 10.1186/1423-0127-21-3 PMC3898375

[b9] Warboys C. M., Amini N., de Luca A. & Evans P. C. The role of blood flow in determining the sites of atherosclerotic plaques. F1000 Med Rep 3, 5 (2011). 21654925 10.3410/M3-5 PMC3096883

[b10] Yamawaki H., Lehoux S. & Berk B. C. Chronic physiological shear stress inhibits tumor necrosis factor–induced proinflammatory responses in rabbit aorta perfused *ex vivo*. Circulation 108, 1619–1625 (2003). 12963644 10.1161/01.CIR.0000089373.49941.C4

[b11] Parmar K. M. . Integration of flow-dependent endothelial phenotypes by Kruppel-like factor 2. J Clin Invest 116, 49–58 (2006). 16341264 10.1172/JCI24787 PMC1307560

[b12] Ohnesorge N. . Erk5 activation elicits a vasoprotective endothelial phenotype via induction of Kruppel-like factor 4 (KLF4). J Biol Chem 285, 26199–26210 (2010). 20551324 10.1074/jbc.M110.103127 PMC2924030

[b13] Walshe T. E., dela Paz N. G. & D’Amore P. A. The role of shear-induced transforming growth factor-β signaling in the endothelium. Arterioscler Thromb Vasc Biol 33, 2608–2617 (2013). 23968981 10.1161/ATVBAHA.113.302161 PMC4129450

[b14] Egorova A. D. . Tgfβ/Alk5 signaling is required for shear stress induced klf2 expression in embryonic endothelial cells. Dev Dyn 240, 1670–1680 (2011). 21604321 10.1002/dvdy.22660

[b15] Yamaguchi K. . Identification of a member of the MAPKKK family as a potential mediator of TGF-beta signal transduction. Science 270, 2008–2011 (1995). 8533096 10.1126/science.270.5244.2008

[b16] Toma I. & McCaffrey T. A. Transforming growth factor-beta and atherosclerosis: interwoven atherogenic and atheroprotective aspects. Cell Tissue Res 347, 155–175 (2012). 21626289 10.1007/s00441-011-1189-3 PMC4915479

[b17] Moonen J. A. J. . Endothelial-to-mesenchymal transition contributes to fibro-proliferative vascular disease and is modulated by fluid shear stress. Cardiovasc Res 108, 377–386 (2015). 26084310 10.1093/cvr/cvv175

[b18] Wink D. A. . Mechanisms of the antioxidant effects of nitric oxide. Antioxid Redox Signal 3, 203–213 (2001). 11396476 10.1089/152308601300185179

[b19] Boon R. A. . KLF2 suppresses TGF-beta signaling in endothelium through induction of Smad7 and inhibition of AP-1. Arterioscler Thromb Vasc Biol 27, 532–539 (2007). 17194892 10.1161/01.ATV.0000256466.65450.ce

[b20] Guo Y. . Kallistatin inhibits TGF-beta-induced endothelial-mesenchymal transition by differential regulation of microRNA-21 and eNOS expression. Exp Cell Res 337, 103–110 (2015). 26156753 10.1016/j.yexcr.2015.06.021 PMC4560618

[b21] Yan F. . Glucagon-like peptide 1 protects against hyperglycemic-induced endothelial-to-mesenchymal transition and improves myocardial dysfunction by suppressing poly(ADP-ribose) polymerase 1 activity. Mol Med 21, 15–25 (2015). 25715248 10.2119/molmed.2014.00259 PMC4461581

[b22] Rassaf T., Feelisch M. & Kelm M. Circulating NO pool: assessment of nitrite and nitroso species in blood and tissues. Free Radic Biol Med 36, 413–422 (2004). 14975444 10.1016/j.freeradbiomed.2003.11.011

[b23] Clark P. R. . MEK5 is activated by shear stress, activates ERK5 and induces KLF4 to modulate TNF responses in human dermal microvascular endothelial cells. Microcirculation 18, 102–117 (2011). 21166929 10.1111/j.1549-8719.2010.00071.x PMC3075844

[b24] Young A. . Flow activation of AMP-activated protein kinase in vascular endothelium leads to Kruppel-like factor 2 expression. Arterioscler Thromb Vasc Biol 29, 1902–1908 (2009). 19696400 10.1161/ATVBAHA.109.193540 PMC2766008

[b25] Sakurai H., Miyoshi H., Toriumi W. & Sugita T. Functional interactions of transforming growth factor beta-activated kinase 1 with IkappaB kinases to stimulate NF-kappaB activation. J Biol Chem 274, 10641–10648 (1999). 10187861 10.1074/jbc.274.15.10641

[b26] Derynck R. & Zhang Y. E. Smad-dependent and Smad-independent pathways in TGF-beta family signalling. Nature 425, 577–584 (2003). 14534577 10.1038/nature02006

[b27] Sorrentino A. . The type I TGF-beta receptor engages TRAF6 to activate TAK1 in a receptor kinase-independent manner. Nat Cell Biol 10, 1199–1207 (2008). 18758450 10.1038/ncb1780

[b28] Sakurai H. Targeting of TAK1 in inflammatory disorders and cancer. Trends Pharmacol Sci 33, 522–530 (2012). 22795313 10.1016/j.tips.2012.06.007

[b29] Yan X., Liu Z. & Chen Y. Regulation of TGF-β signaling by Smad7. Acta Biochim Biophys Sin 41, 263–272 (2009). 19352540 10.1093/abbs/gmp018 PMC7110000

[b30] Hilgarth R. S. . Regulation and function of SUMO modification. J Biol Chem 279, 53899–53902 (2004). 15448161 10.1074/jbc.R400021200

[b31] Bartel D. P. MicroRNAs: genomics, biogenesis, mechanism, and function. Cell 116, 281–297 (2004). 14744438 10.1016/s0092-8674(04)00045-5

[b32] Rassaf T., Bryan N. S., Kelm M. & Feelisch M. Concomitant presence of N-nitroso and S-nitroso proteins in human plasma. Free Radic Biol Med 33, 1590–1596 (2002). 12446216 10.1016/s0891-5849(02)01183-8

[b33] Feelisch M. . Concomitant S-, N-, and heme-nitros(yl)ation in biological tissues and fluids: implications for the fate of NO *in vivo*. FASEB J 16, 1775–1785 (2002). 12409320 10.1096/fj.02-0363com

[b34] Luscinskas F. W. . Cytokine-activated human endothelial monolayers support enhanced neutrophil transmigration via a mechanism involving both endothelial-leukocyte adhesion molecule-1 and intercellular adhesion molecule-1. J Immunol 146, 1617–1625 (1991). 1704400

[b35] Donker R. B. . Absence of *in vivo* generalized pro-inflammatory endothelial activation in severe, early-onset preeclampsia. J Soc Gynecol Investig 12, 518–528 (2005). 10.1016/j.jsgi.2005.06.007 16202929

