# Supplementary material for: Suppression of TAK1 pathway by shear stress counteracts the inflammatory endothelial cell phenotype induced by oxidative stress and TGF-β1
Source: Sci Rep. 2017 Feb 17;7:42487. doi: 10.1038/srep42487 (PMC5314358; doi:10.1038/srep42487)
Supplement: Supplementary Information [file srep42487-s1.pdf]

Supplementary Information

Suppression of TAK1 pathway by shear stress counteracts the inflammatory endothelial cell phenotype induced by oxidative stress and TGF-β1

Ee Soo Lee[1,#], Llorenç Solé Boldo[1], Bernadette O. Fernandez[2], Martin Feelisch[2] & Martin C. Harmsen[1,*]

[1]University of Groningen, University Medical Center Groningen, Department of Pathology and Medical Biology, Groningen, NL-9713 GZ, The Netherlands.

[2]University of Southampton, Southampton General Hospital, Faculty of Medicine, Clinical and Experimental Sciences, Southampton, SO166YD, United Kingdom.

# Present address:

National University of Singapore, Centre for Life Sciences, Department of Physiology, Singapore, 117456, Singapore.

***Corresponding author:**

Prof. Dr. Martin C. Harmsen, PhD

Supplementary materials and methods

Cell culture

HUVEC were maintained in gelatin-coated flasks (0.1% gelatin in distilled water). Trypsin (MP Biomedicals, Illkrich, France) in EDTA was used for the detachment of cells. Cells were seeded at a density of 50, 000 cells/cm^2 on fibronectin-coated (1.5 µg/cm^2; Harbor Bio-Products, MA, USA; 2003 & Alfa Aesar, MA, USA; J64560) plates or slides, left to form a confluent monolayer overnight before treatment. All cell cultures were performed in an incubator at 37°C with 5% CO_2.

Pharmacological inhibition

SB431542 (10 µM; Sigma-Aldrich, Missouri, USA; S4317), 5z-7-oxozeaenol (10 µM; Sigma-Aldrich; O9890), YCG063 (50 µM; Calbiochem, CA, USA; #557354), SB202190 (10 µM; Sigma-Aldrich; S7067) and SC514 (100 µM; Santa Cruz Biotechnology, CA, USA; sc-205504A) were used for inhibition of ALK5, TAK1, ROS production, p38 MAPK and NFκB pathways, respectively. Cells treated with equal volume of dimethyl sulfoxide (DMSO) served as vehicle controls.

Leukocyte-endothelium adhesion assay

Confluent monolayer of HUVEC were stimulated for 48 h with or without 10 ng/ml citric acid activated-TGF-β1 (Peprotech, NJ, USA; #100-21C) in RPMI 1640 basal medium, supplemented with 20% heat-inactivated foetal bovine serum, 2 mM L-glutamine, 5 U/ml heparin and 1% penicillin-streptomycin. Cells treated with endothelial cell medium for 48 h were harvested as unstimulated controls. Cells stimulated with IL-1β (10 ng/ml) for 48 h in endothelial cell medium served as a positive control. Human myeloid leukaemia cell line, HL60 (European Collection of Authenticated Cell Cultures, ECACC 98070106, UK) was stimulated with IL-1β (10 ng/ml) for 6 h in RPMI 1640 basal medium, supplemented with 10% heat-inactivated foetal bovine serum. Stimulated HL60 were washed with RPMI 1640 basal medium and incubated with Vybrant® CM-Dil Cell Labelling Solutions (Molecular Probes, Leiden, The Netherlands) for 30 min. Labelled HL60 were added onto HUVEC monolayer and incubated for one hour. All stimulations and incubations were performed in

an incubator at 37°C with 5% CO_2. After incubation, each culture well were washed twice with PBS and fixed for 30 min with 4% paraformaldehyde containing 4',6-diamidino-2-phenylindole (DAPI; Sigma-Aldrich, MA, USA) at room temperature. Each culture well was examined with an inverted microscope (DM IL; Leica Microsystems, Wetzlar, Germany). Images were acquired using a digital microscope camera Leica DFC425C (Leica Microsystems). The total number of adhered leukocytes over the number of HUVEC (n > 100) in representative fields was calculated. Data of each experimental condition are presented as fold changes relative to the unstimulated control.

ROS measurement

HUVEC were washed with RPMI 1640 basal medium and detached with accutase (Sigma-Aldrich). Cell pellets were re-suspended in RPMI 1640 basal medium consisted of 20 μM of 2',7'-dichloroflorescein diacetate (Sigma-Aldrich; D6883) and incubated for 30 min at 37°C with 5% CO_2. The fluorescent products of 2',7'-dichloroflorescein oxidation by hydrogen peroxide (H_2O_2), peroxynitrite (ONOO-), hydroxyl radicals (•OH) or superoxide anions ($O_2^{\bullet -}$) indicate the level of intracellular ROS formation[1]. Data analysis was performed with Kaluza® Flow Analysis Software version 1.3 (Beckman Coulter Inc, California, USA).

RT-qPCR

One μg of total RNA was reverse-transcribed using the FirstStrand cDNA synthesis kit (Fermentas UAB, Lithuania) according to the manufacturer's protocol. The cDNA-equivalent of 5 ng RNA was used for amplification in 384-well microtitre plates in a TaqMan ABI7900HT cycler (Applied Biosystems, CA, USA) in a final reaction volume of 10 μl containing 5μl SYBR Green mix with ROX (Bio-Rad Laboratories, CA, USA or Roche, IN, USA) and 0.5 μl primers mix (Biolegio, Nijmegen, The Netherlands or Sigma-Aldrich). All cDNA samples were amplified in duplicate. Cycle threshold (Ct) values for individual reactions were determined using ABI Prism SDS 2.2 data processing software (Applied Biosystems). The following formulas were employed for calculation of relative fold change for gene expression in different experimental conditions, $2^{-\Delta\Delta Ct(gene)}$: Ct-values of gene of interest were first normalized against geometric mean of housekeeping genes, *B2M* and

GAPDH expression by the following equation [ΔCt(gene) = Ct(gene) – Ct(geometric mean of *B2M* and *GAPDH*)]. Next, relative gene expression levels, ΔΔCt(gene) were calculated as follows: [ΔΔCt(gene) = ΔCt(gene) of samples from different experimental condition – average of ΔCt(gene) of controls]. Lastly, relative fold change for gene expression in samples were calculated as $2^{-\Delta\Delta Ct(gene)}$.

Immunofluorescent staining

Treated cells on diagnostic slides (VWR International, Amsterdam, The Netherlands) were washed with chilled PBS and fixed at room temperature with 2% paraformaldehyde for 15 min. Fixed cells were rehydrated with PBS for 10 min, followed by a 10-min-permeabilisation with 0.5% Triton X-100 (Sigma Aldrich) and a 10-min-blocking with 10% donkey serum. Primary antibodies incubation was performed in 10% donkey serum at 4°C overnight. Then, cells were washed with 0.1% Tween-20 in PBS and incubated with Alexa Fluor® 488-conjugated donkey anti-mouse IgG or Alexa Fluor® 555-conjugated donkey anti-rabbit IgG (1:200; Life Technologies, CA, USA) in PBS containing DAPI (Sigma-Aldrich) and 10% normal human serum at room temperature for one hour. Slides were mounted with Citifluor AP1 (Agar Scientific, Stansted, UK). Images of staining were captured with the PCO/ Pixelfly II camera (PCO AG, Kelheim, Germany).

Immunoblotting

Treated cells were washed with chilled PBS. Whole cell lysates were prepared in radio-immunoprecipitation assay (RIPA) buffer (Thermo Scientific, IL, USA) supplemented with 1% protease inhibitor cocktail and 1% phosphatase inhibitor cocktail (both Sigma-Aldrich). Sonicated cell lysates (50 μg/lane) were separated by gel electrophoresis in a 10% denaturing SDS-polyacrylamide gel and subsequently blotted onto nitrocellulose membrane (Hybond-P; Amersham Pharmacia Biotech, England, UK) according to standard protocols. Blots were blocked for one hour in Odyssey Blocking Buffer (Li-COR Biosciences, Nebraska, USA) at room temperature and incubated at 4°C overnight with primary antibodies in Odyssey Blocking Buffer, supplemented with 0.1% Tween-20. Subsequently, blots were washed with 0.1% Tween-20 in Tris-buffered saline and incubated with secondary antibodies, *i.e.* goat anti-rabbit conjugated with IRDye700 or goat anti-mouse conjugated

with IRDye800 (both 1:10 000; Li-COR Biosciences). The blots were scanned using an Odyssey Infrared Imaging System (Li-COR Biosciences). The intensity of bands was quantified using ImageJ version 1.47 (National Institute of Health, Bethesda, MD, USA).

References

1. Eruslanov, E. & Kusmartsev, S. Identification of ROS using oxidized DCFDA and flow-cytometry in *Advanced protocols in oxidative stress II* (ed. Armstrong, D.) 57-72 (Humana Press, 2010).

Supplementary Table S1. Primer sequences of genes.

gene		primer Sequence (5' – 3')
B2M	F	TGCTGTCTCCATGTTTGATGTATCT
	R	TCTCTGCTCCCCACCTCTAAGT
GAPDH	F	AGCCACATCGCTCAGACAC
	R	GCCCAATACGACCAAATCC
SELE	F	ACCAGCCCAGGTTGAATG
	R	GGTTGGACAAGGCTGTGC
ICAM1	F	CCTTCCTCACCGTGTACTGG
	R	AGCGTAGGGTAAGGTTCTTGC
VCAM1	F	TGGACATAAGAAACTGGAAAAGG
	R	CCACTCATCTCGATTTCTGGA
CXCL8	F	CTTTCAGAGACAGCAGAGCA
	R	ACACAGAGCTGCAGAAATCA
CCL2	F	AGTCTCTGCCGCCCTTCT
	R	GTGACTGGGGCATTGATTG
PECAM1	F	GCAACACAGTCCAGATAGTCGT
	R	GACCTCAAACTGGGCATCAT
THBD	F	AATTGGGAGCTTGGGAATG
	R	TGAGGACCTGATTAAGGCTAGG
NOS3	F	ATTTCCACGGAAACTACAGG
	R	TCTCCCTAAGCTGGTAGGTG
ACTA2	F	CTGTTCCAGCCATCCTTCAT
	R	TCATGATGCTGTTGTAGGTGGT
TAGLN	F	CAAAGCCATCAGGGTCCTC
	R	TTCCAGACTGTTGACCTCTTTG
CNN1	F	TGAAGTACGCAGAGAAGCAG
	R	CAGCTTGGGGTCGTAGAG
TNFA	F	CTACTCCCAGGTCCTCTTCA
	R	GTTGACCTTGGTCTGGTAGG
IL1B	F	AAGCTGGAATTTGAGTCTGC
	R	ACACAAATTGCATGGTGAAG
IL6	F	ACTTGCCTGGTGAAAATCAT
	R	CAGGAACTGGATCAGGACTT
SMAD6	F	TGCAACCCCTACCACTTCA
	R	CGAGGAGACAGCCGAGAGT
SMAD7	F	CGATGGATTTTCTCAAACCAA
	R	AGGGGCCAGATAATTCGTTC

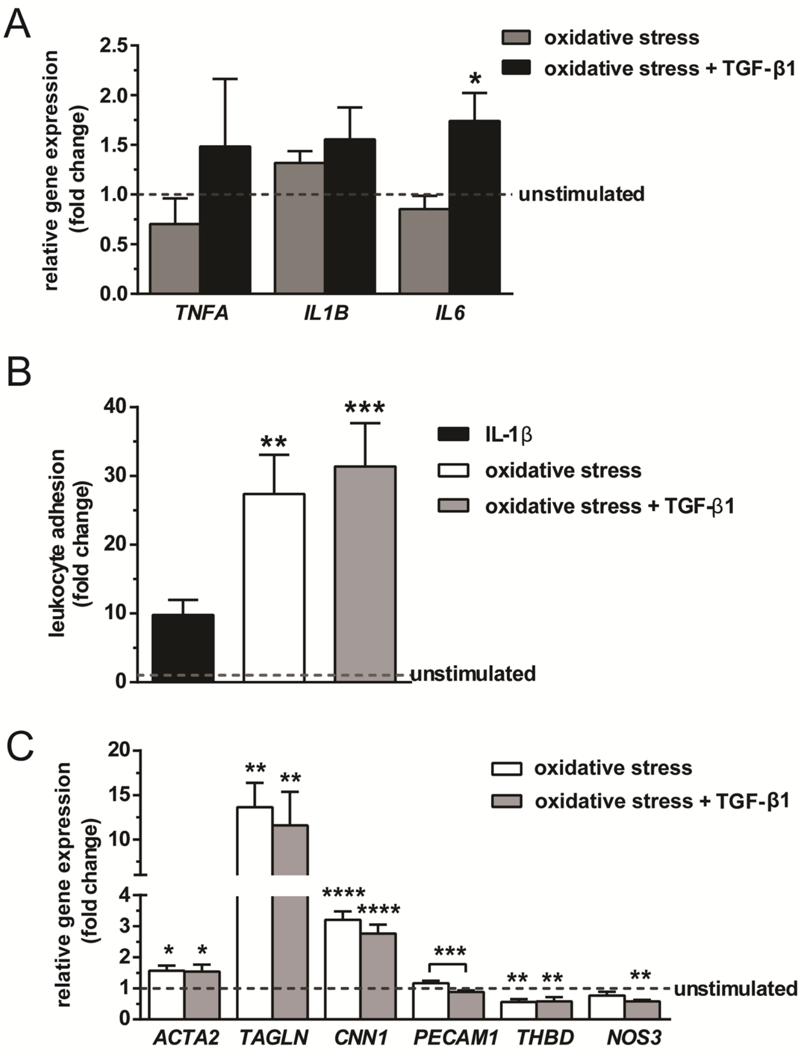

Supplementary Figure S1: The effect of oxidative stress and TGF-β1 stimulation on *TNFA, IL1B* and *IL6* expression, leukocyte interaction with endothelial cells and endothelial-to-mesenchymal transition (EndMT). (**A**) As compared with unstimulated condition (shown as a dotted line), oxidative stress has no effect on the gene expression of *TNFA*, *IL1B* and *IL6,* but the combined effect of oxidative stress and TGF-β1 up-regulates the expression of *IL6* (N=3). (**B**) Endothelial cells stimulated by oxidative stress and TGF-β1 show higher interaction with leukocytes than the unstimulated control (dotted line). Endothelial cells stimulated with pro-inflammatory cytokine, IL-1β serves as a positive control for the assay (N=3). (**C**) Oxidative stress and TGF-β1 upregulate the gene expression of mesenchymal markers (*ACTA2, TAGLN* and *CNN1*), but downregulate the gene expression of endothelial markers (*PECAM1, THBD* and *NOS3*) as compared with the unstimulated control (dotted line; N=3). *p<0.05, **p<0.01, ***p<0.001 & ****p<0.0001.

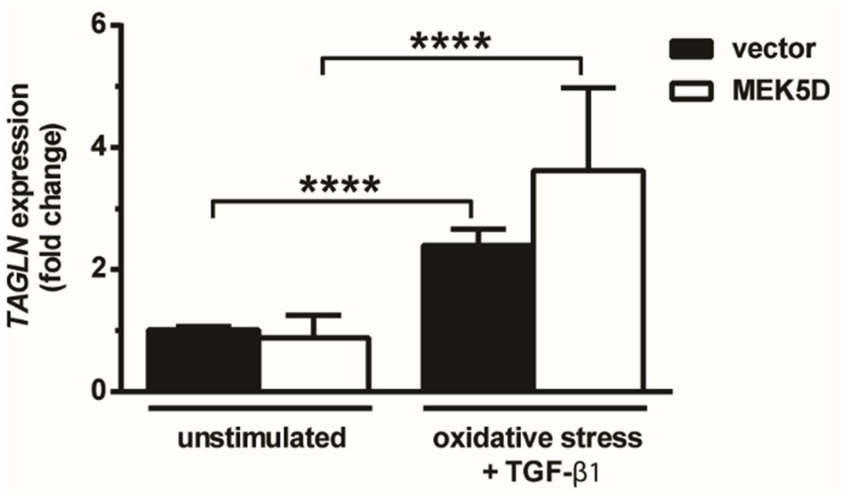

Supplementary Figure S2: Constitutive activation of ERK5 signalling increases the expression of mesenchymal marker, *TAGLN* upon TGF-β1 stimulation in MEK5D-transduced cells (N=3). ****P<0.0001.

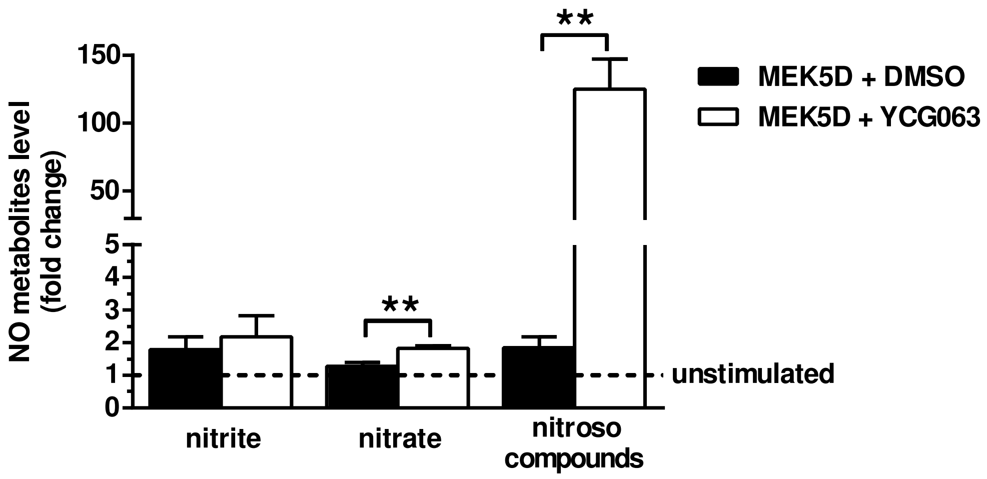

Supplementary Figure S3: YCG063 increases the generation of nitrate and nitroso compounds in MEK5D-transduced cells upon oxidative stress and TGF-β1 stimulation, but has no effect on nitrite formation (dotted line represents unstimulated condition; N=3). **p<0.01.

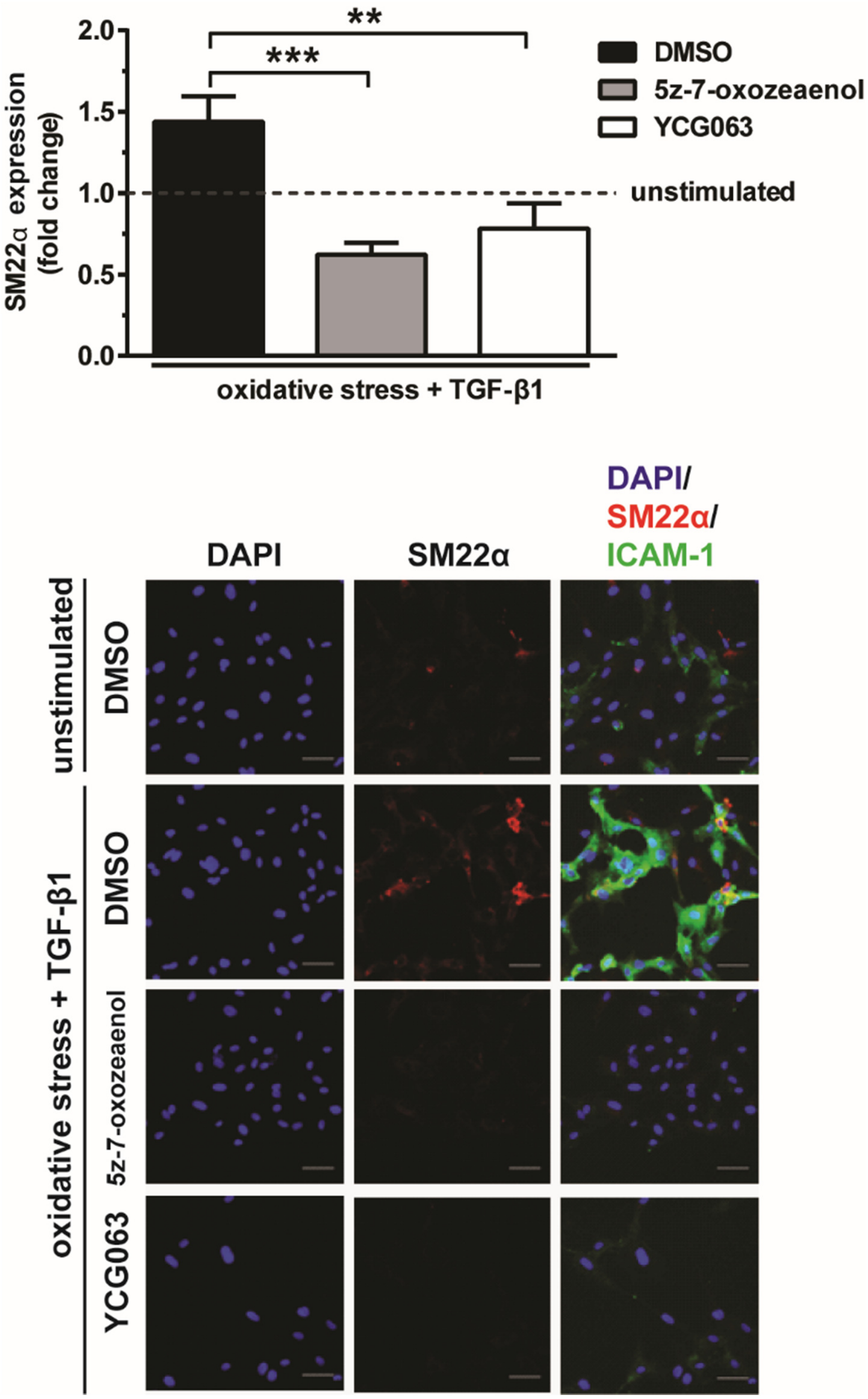

Supplementary Figure S4: TAK1 pathway and ROS contribute to induction of EndMT while exerting inflammatory effects on endothelial cells. Mesenchymal protein, SM22α co-expressed with ICAM-1 upon oxidative stress and TGF-β1 stimulation. Treatment with either 5z-7-oxozeaenol or YCG063 suppresses the effects of oxidative stress and TGF-β1 on upregulating the expression of SM22α (N=3). **p<0.01 & ***p<0.001.
